# The Value of MicroRNA-375 Detection for Triaging Primary Human Papillomavirus Positive Women: A Cross-Sectional Study in a General Population

**DOI:** 10.3389/fonc.2021.771053

**Published:** 2021-10-28

**Authors:** Qiongyan Wu, Lingfang Wang, Xiumin Zhao, Qifang Tian, Fenfen Wang, Ni Sima, Liqian Qiu, Weiguo Lu, Xing Xie, Xinyu Wang, Xiaodong Cheng

**Affiliations:** ^1^ Department of Women Health, Women’s Hospital, School of Medicine, Zhejiang University, Hangzhou, China; ^2^ Key Laboratory of Women’s Reproductive Health of Zhejiang Province, Women's Hospital, School of Medicine, Zhejiang University, Hangzhou, China; ^3^ Department of Gynecologic Oncology, Taizhou First People’s Hospital, Taizhou, China; ^4^ Department of Gynecologic Oncology, Women’s Hospital, School of Medicine, Zhejiang University, Hangzhou, China

**Keywords:** cervical cancer screening, microRNA, human papillomavirus, cervical cytology, cervical intraepithelial neoplasia

## Abstract

**Purpose:**

This study aims to validate the value of microRNA (miRNA) detection for triaging human papillomavirus (HPV)-positive women in the general population.

**Patients and Methods:**

miR-375 detection in cervical exfoliated cells has been demonstrated to have the superior value to cytology in triaging primary HPV-positive women in the hospital population. In this study, residual samples of cervical exfoliated cells from 10,951 women in a general population were used to detect miRNA. The performance efficiency of miRNA detection in identifying high-grade cervical intraepithelial neoplasia (CIN) was evaluated. Pearson chi-square test and McNemar pairing test were used to compare miRNA detection and cytology.

**Results:**

In valid 9,972 women aged 25–65, miR-375 expression showed a downward trend along with an increase in cervical lesion severity. The expression level of miR-375 ≤1.0 × 10^-3^ was identified as positive. In the HPV-positive and 12 HPV genotypes other than 16/18 (HR12)-positive women, miR-375 detection showed equivalent sensitivity, specificity, positive predictive value (PPV), and negative predictive value (NPV) to that of cytology (≥ASC-US) and higher or similar sensitivity and NPV but lower specificity and PPV than that of cytology (≥ASC-H) in identifying CIN3+ and CIN2+. In HPV 16-positive women, miR-375 positivity had higher sensitivity and NPV but lower specificity and PPV than that of cytology (≥ASC-H and HSIL) in identifying CIN3+ and CIN2+. The immediate CIN3+ risk of miR-375 positivity was 19.8% (61/308) in HPV-positive, 10.8% (22/204) in HR12-positive, and 43.5% (37/85) in HPV16-positive women, respectively.

**Conclusion:**

The detection of miR-375 in cervical exfoliated cells may be an optional method for triaging primary HPV-positive women in population-based cervical cancer screening.

## Introduction

Cervical cancer remains one of the most common malignancies in women worldwide (1). According to the results released by the International Agency for Research on Cancer, nearly 570,000 women are estimated to develop cervical cancer worldwide, with more than 310,000 deaths (2).

Population-based screening, using human papillomavirus (HPV) test and cytology, is one of the most effective prevention strategies for cervical cancer (3, 4). In 2008, the European Research Organization on Genital Infection and Neoplasia proposed the scheme of primary HPV screening (5). In 2015, the American Society of Colposcopy and Cervical Pathology (ASCCP) and the Society of Gynecologic Oncology issued interim guidance that recommended the Food and Drug Administration-approved HPV test to be used for the primary screening of cervical cancer in women over 25 years of age as an alternative to cytology-based examination (6). In 2018, the US Preventive Services Task Force conducted a systematic literature review, stating that primary HPV screening had a higher cervical intraepithelial lesion (CIN) 3+ detection rate than cytology, and a 5-year round of primary HPV screening showed the best risk–benefit balance (7). The 2019 ASCCP Risk-Based Management Consensus Guideline for Abnormal Cervical Cancer Screening Tests and Cancer Precursors stated that HPV-based testing was the basis for risk estimation (8).

In view of the low positive predictive value (PPV) of the HPV test, HPV-positive women should be triaged, and cytology is currently preferred worldwide (9–11). However, the inherent disadvantage of cytology is its low sensitivity (12). Moreover, cytology examination depends on cytologists, who are usually lacking in developing countries. Cytology can be used as a triage method only in settings where cytology is of high quality. Therefore, the development of a new method, other than cytology, to triage HPV-positive women is imperative.

MicroRNAs (miRNAs) are some single-stranded RNAs, and they are only 19–25 nucleotides in length. miRNAs are non-coding RNAs, but they regulate gene expression mainly by binding to sequence motifs located within the 3′ untranslated region (UTR) of mRNA transcripts (13). Numerous studies have shown that the expressions of some miRNAs were dysregulated during the development of cancers, and miRNA detection can be used as a tumor biomarker (14, 15). In our previous research, we detected 875 human miRNAs in cervical cancer and normal cervical tissues through chip technology and found that 14 miRNAs (including miR-375 and miR-424) were down-regulated in cervical cancer (16). Then, the efficiency of miR-375 and miR-424 detection was further confirmed to be superior to that of cytology in cervical exfoliated cells for triaging HPV-positive women in gynecological clinics (17). However, the previous study was derived from a clinic-based population, so it might possess biases such as higher HPV-positive proportion in recruited women and higher abnormal cytology and histology proportion in HPV-positive women who actively visited the outpatient clinic. For the general population who is the main target of cervical cancer screening, the values of miR-375 and miR-424 detection for triaging HPV-positive women have not been studied and confirmed.

To further validate the value of miRNA detection for triaging HPV-positive women in the general population, we utilized residual cervical exfoliated cell specimens from a population-based cervical cancer screening program, detected the expression of miR-375 and miR-424, and evaluated the performance efficiency of miR-375 and miR-424 detection to identify high-grade CIN in HPV-positive women, with histological diagnosis as the gold standard and cytology as the control. The aim of this study is to provide a new method for HPV-based cervical cancer screening in the general population.

## Materials and Methods

### Subject Recruitment and Sample Collection

**Figure 1:**
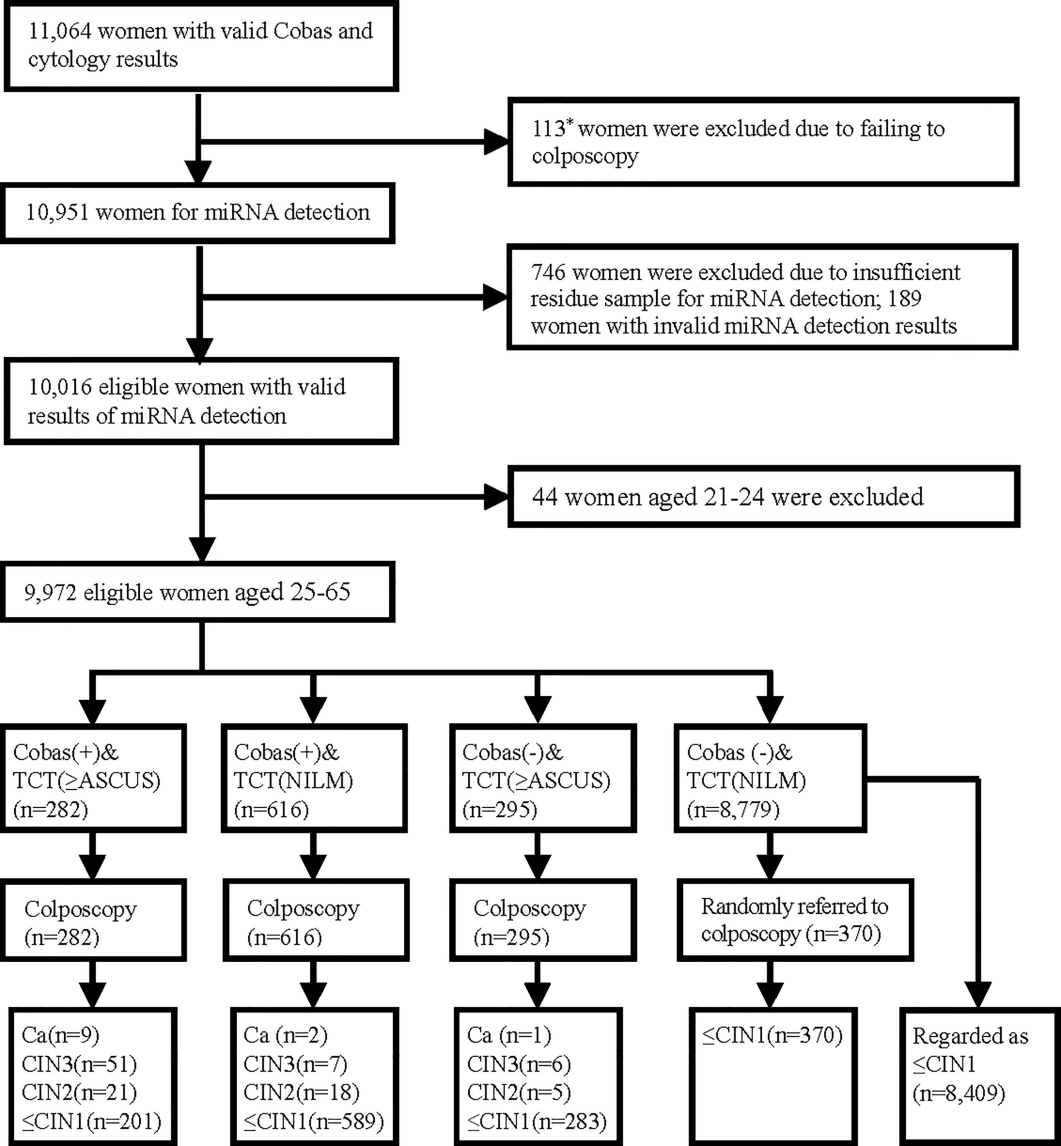
Test results and outcomes. *Included 86 women who had abnormal Cobas HPV/cytology results but failed to have colposcopy and 27 women with normal Cobas HPV and cytology results who were randomly selected for colposcopy but did not undergo colposcopy.

In 2015, 11,356 women aged 21–65 years participated in a cervical cancer screening program in Longyou County, Zhejiang Province, China. The detailed program design and inclusion/exclusion criteria for participants were described in our previous paper (18). In this study, we obtained residual cervical exfoliated cell samples from eligible women who had valid HPV and cytology test results. The flow chart is shown in **Figure 1**. All women signed informed consent forms.

The study was in accordance with the 2013 Declaration of Helsinki and was approved by the Ethics Committee of Women’s Hospital, School of Medicine, Zhejiang University.

### HPV Test and Cytology Examination

HPV DNA was tested using the Cobas HPV Test (Cobas^®^ 4800 Test, Roche Molecular Systems, Pleasanton, CA, USA), and cytology was examined using the Thinprep cytology test (Hologic, Bedford, MA, USA). The Cobas HPV results were divided into HPV-negative, HPV 16/18-positive (test positive for either genotype 16 or 18, with or without 12 other genotypes), and high-risk 12 (HR12)-positive (result was negative for genotypes 16 and 18 but positive for one or more of the 12 other high-risk genotypes, including HPV 31, 33, 35, 39, 45, 51, 52, 56, 58, 59, 66, and 68). The cytology results were reported according to the Bethesda 2014 classification (19). The cytology diagnoses were divided into negative for intraepithelial lesion or malignancy, atypical cells of undetermined significance (ASC-US), low-grade squamous intraepithelial lesion (LSIL), atypical squamous cells—cannot exclude high-grade squamous intraepithelial lesion (ASC-H), high-grade squamous intraepithelial lesion (HSIL), atypical glandular cells (AGC), and cervical cancer cells.

### miRNA Detection

The expression of miR-375 and miR-424 in cervical exfoliated cells was detected in the same way as in our previous study (16, 17). Total RNA containing miRNAs from each sample was extracted with TRIzol Reagent (Invitrogen, Carlsbad, CA, USA). Stem-loop real-time RT-qPCR was used for miRNA detection. cDNA was synthesized from 0.5 μg of total RNA in a 10-μl reaction volume with the PrimeScript RT Reagent Kit (TaKaRa, Dalian, China), and the reverse transcription (RT) reaction program was as follows: 30 min at 16°C, 30 min at 42°C, 5 min at 85°C, and a final temperature of 4°C. qPCR was performed to quantify the expression of miR-375 and miR-424 using an SYBR Premix Ex Taq kit (TaKaRa, Dalian, China) on an Applied Biosystems 7900HT fast real-time system (Applied Biosystems, Foster City, CA, USA). One microliter (μl) of the RT product was added into a total reaction volume of 20 μl, and the reactions were incubated in a 96-well plate at 95°C for 30 s, followed by 40 cycles of 95°C for 5 s and 60°C for 30 s. U6 was used as an endogenous control for normalization. The relative quantitative method was used, and the relative expression level of miRNA was calculated based on the following equation: *F* = 2^-ΔCt^, where ΔCt = Ct (miRNA) – Ct (U6). A high *F* value indicates a higher relative expression level of miR-375 or miR-424. Primers are shown in **Supplementary Table S1** (available online). A total of 10,016 samples showed valid results of miRNA detection.

### Colposcopy and Histological Diagnosis

A total of 1,193 women with abnormal cytology (ASC-US or worse) or positive HPV results were referred to colposcopy with or without biopsy; meanwhile, 4.5% of 8,806 women with both negative cytology and HPV results were randomly selected for colposcopy, and 370 women actually underwent colposcopy. The histological diagnosis standard was the 2014 WHO Classification of Tumors of the Female Genital Tract, with diagnoses classified as follows: CIN1 or better, CIN2, CIN3, and invasive cancer (20). For ethical reasons, most women with both negative HPV and cytology results were not referred to colposcopy but were regarded as CIN1 or better. Cobas HPV test, cytology examination, miRNA detection, and histological diagnosis results were all blinded to each other. The colposcopy specialists were aware of the Cobas HPV detection and cytology results but did not know the miRNA results. All women with abnormal histological diagnoses were treated according to the ASCCP 2013 guidelines.

### Statistical Analysis

SPSS software (version 19.0; SPSS Inc., Chicago, IL, USA) and MedCalc software (version 15.6; MedCalc Inc., Ostend, Belgium) were used for the statistical analysis. *P <*0.05 (bilateral) was considered statistically significant. A linear regression analysis was used to analyze the difference of miRNA expression among different groups of cervical lesion. Pearson chi-square test, Fisher’s exact test, and McNemar pairing test were performed to compare the various rates. The cutoff values for miRNA detection in the diagnosis of high-grade CIN (CIN2+) were determined according to the receiver operating characteristic (ROC) curve and maximum Youden index.

## Results

### Population Profile and Determination of miRNA Cutoff Values

As shown in **Figure 1**, 9,972 women aged 25–65 obtained valid results of the Cobas HPV, cytology, and miRNA testing. The final cervical histological results included 120 women with CIN2+ (12 women with invasive cancer, 64 with CIN3, and 44 with CIN2) and 9,852 women with CIN1 or better.

**Figure 2:**
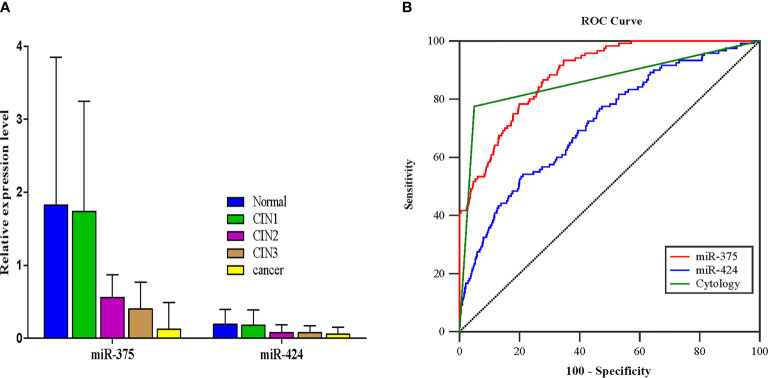
The results of miR-375 and miR-424 detection and the determination of the cutoff value for identifying CIN2+. **(A)** The expression of miR-375 and miR-424 in different pathological results, median (IQR) (*P* < 0.001, *P* < 0.001). **(B)** The receiver operating characteristic curves of miRNA detection and cytology (≥ASC-US) for identifying CIN2+.

The relative expression levels of miR-375 and miR-424 showed a downward trend along with an increase in cervical lesion severity (*P* < 0.001, *P* < 0.001) (**Figure 2A**). Based on the sensitivity and specificity for predicting CIN2+, ROC curves were generated. The areas under the curve (AUCs) of the miR-375 test, miR-424 test, and cytology (≥ASC-US, including ASC-US, LSIL, ASC-H, HSIL, AGC, and cancer cell) to predict CIN2+ were 0.887, 0.716, and 0.863, respectively, and miR-375 showed the greatest AUC among them (**Figure 2B**). According to the maximum Youden index, the cutoff values of miR-375 and miR-424 to identify CIN2+ in 9,972 subjects were 0.999 × 10^-3^ and 0.796 × 10^-5^, respectively. When the test result of miR-375 was less than or equal to 1.0 × 10^-3^, it was defined as positive; when the test result was higher than 1.0 × 10^-3^, it was defined as negative. Since the AUC for miR-424 was less than that for cytology, a subsequent analysis of miR-424 was not conducted.

### The Value of miR-375 in Identifying High-Grade CIN in the HPV-Positive Population

A total of 898 women tested positive for HPV, among whom 69 women were CIN3 or worse (CIN3+), 39 were CIN2, and 790 were CIN1 or better. To identify CIN3+, the sensitivity, specificity, PPV, and NPV of miR-375 relative detection were not significantly different from those of cytology (≥ASC-US) (*P* > 0.05). However, miR-375 detection had significantly higher sensitivity (88.4 *vs*. 68.1%, *P* = 0.003) and no difference in NPV but had significantly lower specificity and PPV (70.2 *vs*. 96.4%, *P* < 0.001; 19.8 *vs*. 61.0%, *P* < 0.001, respectively) than cytology (≥ASC-H, including ASC-H, HSIL, and cancer cell). To identify CIN2+, miR-375 detection positivity had significantly higher sensitivity (88.0 *vs*. 75.0%, *P* = 0.018) and NPV (97.8 *vs*. 95.6%, *P* = 0.035) and not different specificity and PPV than cytology (≥ASC-US). Additionally, miR-375 detection had significantly higher sensitivity (88.0 *vs*. 53.7%, *P* < 0.001) and NPV (97.8 *vs*. 93.9%, *P* < 0.001) but had significantly lower specificity and PPV (73.0 *vs*. 97.6%, *P* < 0.001; 30.8 *vs*. 75.3%, *P* < 0.001, respectively) than cytology (≥ASC-H), as shown in **Table 1**.

**Table 1:** Comparison of performance efficiency among miR-375 detection and cytology tests for identifying CIN3+ and CIN2+ in HPV-positive women.

Test	Sensitivity		Specificity		PPV		NPV	
	*n*/*N* (%, 95% CI)	*P*	*n*/*N* (%, 95% CI)	*P*	*n*/*N* (%, 95% CI)	*P*	*n*/*N* (%, 95% CI)	*P*
CIN3+								
miR-375	61/69 (88.4%, 78.4–94.9)		582/829 (70.2%, 67.0–73.3)		61/308 (19.8%, 15.5–24.7)		582/590 (98.6%, 97.4–99.4)	
Cytology (≥ASC-US)	60/69 (87.0%, 76.7–93.9)	1.000	607/829 (73.2%, 70.1–76.2)	0.195	60/282 (21.3%, 16.7–26.5)	0.658	607/616 (98.5%, 97.2–99.3)	0.877
Cytology (≥ASC-H)	47/69 (68.1%, 55.8–78.8)	0.003^**^	799/829 (96.4%, 94.9–97.6)	<0.001^**^	47/77 (61.0%, 49.3–72.0)	<0.001^**^	799/821 (97.3%, 96.0–98.3)	0.089
CIN2+								
miR-375	95/108 (88.0%, 80.3–93.4)		577/790 (73.0%, 69.8–76.1)		95/308 (30.8%, 25.7–36.3)		577/590 (97.8%, 96.3–98.8)	
Cytology (≥ASC-US)	81/108 (75.0%, 65.8–82.8)	0.018^*^	589/790 (74.6%, 71.4–77.6)	0.542	81/282 (28.7%, 23.5–34.4)	0.574	589/616 (95.6%, 93.7–97.1)	0.035^*^
Cytology (≥ASC-H)	58/108 (53.7%, 43.9–63.4)	<0.001^**^	771/790 (97.6%, 96.3–98.6)	<0.001^**^	58/77 (75.3%, 64.2–84.4)	<0.001^**^	771/821 (93.9%, 92.1–95.5)	<0.001^**^

CIN3+, cervical intraepithelial neoplasia grade 3 or worse; CIN2+, cervical intraepithelial neoplasia grade 2 or worse; CI, confidence interval; PPV, positive predictive value; NPV, negative predictive value.

The relative expression level of miR-375 was compared with cytology (≥ASC-US) and cytology (≥ASC-H) separately.

*P < 0.05, **P < 0.01.

In the HPV-positive population, the immediate CIN3+ risk was 19.8% (61/308) for miR-375 detection, 21.3% (60/282) for cytology (≥ASC-US), and 61.0% (47/77) for cytology (≥ASC-H).

### The Value of miR-375 in Identifying High-Grade CIN in the HR12-Positive Population

A total of 681 women were HR12-positive, among whom 26 women were CIN3+, 21 women were CIN2, and 634 women were CIN1 or better. To identify CIN3+ and CIN2+, the sensitivity, specificity, PPV, and NPV of miR-375 detection were not significantly different from those of cytology (≥ASC-US). However, compared with cytology (≥ASC-H), miR-375 detection had a higher, but not significantly higher, sensitivity and NPV, while it had a significantly lower specificity and PPV (72.2 *vs*. 96.8%, *P* < 0.001; 10.8 *vs*. 46.2%, *P* < 0.001, respectively) for identifying CIN3+. For identifying CIN2+, the efficacy of miR-375 detection was similar to that in identifying CIN3+, except for a significantly higher sensitivity (83.0 *vs*. 51.1%, *P* = 0.002), as shown in **Table 2**.

**Table 2:** Comparison of performance efficiency among miR-375 and cytology tests for detecting CIN3+ and CIN2+ in HPV non-16/18+ women.

Test	Sensitivity		Specificity		PPV		NPV	
	*n*/*N* (%, 95% CI)	*P*	*n*/*N* (%, 95% CI)	*P*	*n*/*N* (%, 95% CI)	*P*	*n*/*N* (%, 95% CI)	*P*
CIN3+								
miR-375	22/26 (84.6%, 65.1–95.6)		473/655 (72.2%, 68.6.1–75.6)		22/204 (10.8%, 6.9–15.9)		473/477 (99.2%, 97.9–99.8)	
Cytology (≥ASC-US)	23/26 (88.5%, 69.9–97.6)	1.000	479/655 (73.1%, 69.6–76.5)	0.757	23/199 (11.6%, 7.5–16.8)	0.805	479/482 (99.4%, 98.2–99.9)	0.694
Cytology (≥ASC-H)	18/26 (69.2%, 48.2–85.7)	0.289	634/655 (96.8%, 95.1–98.0)	<0.001^**^	18/39 (46.2%, 30.1–62.8)	<0.001^**^	634/642 (98.8%, 97.6–99.5)	0.513
CIN2+								
miR-375	39/47 (83.0%, 69.2–92.4)		469/634 (74.0%, 70.4–77.4)		39/204 (19.1%, 14.0–25.2)		469/477 (98.3%, 96.7–99.3)	
Cytology (≥ASC-US)	36/47 (76.6%, 62.0–87.7)	0.549	471/634 (74.3%, 70.7–77.7)	0.950	36/199 (18.1%, 13.0–24.2)	0.791	471/482 (97.7%, 96.0–98.9)	0.501
Cytology (≥ASC-H)	24/47 (51.1%, 36.1–66.0)	0.002^**^	619/634 (97.6%, 96.1–98.7)	<0.001^**^	24/39 (61.5%, 44.6–76.6)	<0.001^**^	619/642 (96.4%, 94.7–97.7)	0.055

miR-375 was compared with cytology (≥ASC-US) and cytology (≥ASC-H) separately.

**P < 0.01.

In the HR12-positive women, the immediate CIN3+ risk was 10.8% (22/204) for miR-375 positivity and 46.2% (18/39) for cytology (≥ASC-H).

### The Value of miR-375 in Triaging the HPV16-Positive Population for Expedited Treatment

A total of 162 women were HPV16-positive, among whom 41 women were CIN3 or worse, 16 women were CIN2, and 105 women were CIN1 or better. In the HPV16-positive population, miR-375 detection had significantly higher sensitivity (90.2 *vs*. 70.7%, *P* = 0.022), not significantly different NPV, and significantly lower specificity and PPV (60.3 *vs*. 92.6%, *P* < 0.001; 43.5 *vs*. 76.3%, *P* = 0.001, respectively) than cytology (≥ASC-H) for predicting CIN3+. Compared with cytology (≥HSIL), miR-375 detection had a significantly higher sensitivity and NPV (90.2 *vs*. 51.2%, *P* < 0.001; 94.8 *vs*. 85.4%, *P* = 0.036, respectively) but significantly lower specificity and PPV (60.3 *vs*. 96.7%, *P* < 0.001; 43.5 *vs*. 84.0%, *P* < 0.001, respectively). When predicting CIN2+, the results were similar to those for predicting CIN3+, as shown in **Table 3**.

**Table 3:** Comparison of performance efficiency among miR-375 and cytology tests for detecting CIN3+ and CIN2+ in HPV16+ women.

Test	Sensitivity		Specificity		PPV		NPV	
	*n*/*N* (%, 95% CI)	*P*	*n*/*N* (%, 95% CI)	*P*	*n*/*N* (%, 95% CI)	*P*	*n*/*N* (%, 95% CI)	*P*
CIN3+								
miR-375	37/41 (90.2%, 76.9–97.3)		73/121 (60.3%, 51.0–69.1)		37/85 (43.5%, 32.8–54.7)		73/77 (94.8%, 87.2–98.6)	
Cytology (≥ASC-H)	29/41 (70.7%, 54.5–83.9)	0.022^*^	112/121 (92.6%, 86.4–96.5)	<0.001^**^	29/38 (76.3%, 59.8–88.6)	0.001^**^	112/124 (90.3%, 83.7–94.9)	0.254
Cytology (≥HSIL)	21/41 (51.2%, 35.1–67.1)	<0.001^**^	117/121 (96.7%, 91.8–99.1)	<0.001^**^	21/25 (84.0%, 63.9–95.5)	<0.001^**^	117/137 (85.4%, 78.4–90.9)	0.036^*^
CIN2+								
miR-375	52/57 (91.2%, 80.7–97.1)		72/105 (68.6%, 58.8–77.3)		52/85 (61.2%, 50.0–71.6)		72/77 (93.5%, 85.5–97.9)	
Cytology (≥ASC-H)	34/57 (59.7%, 45.8–72.4)	<0.001^**^	101/105 (96.2%, 90.5–99.0)	<0.001^**^	34/38 (89.5%, 75.2–97.1)	0.002^**^	101/124 (81.5%, 73.5–87.9)	0.016^*^
Cytology (≥HSIL)	24/57 (42.1%, 29.1–55.9)	<0.001^**^	104/105 (99.1%, 94.8–99.9)	<0.001^**^	24/25 (96.0%, 79.7–99.9)	0.001^**^	104/137 (75.9%, 67.9–82.8)	0.001^*^

miR-375 was compared with cytology (≥ASC-H) and cytology (≥HSIL) separately.

*P < 0.05, **P < 0.01.

In HPV16-positive women, the immediate CIN3+ risk was 43.5% (37/85) for miR-375 positivity, 76.3% (29/38) for cytology (≥ASC-H), and 84.0% (21/25) for cytology (≥HSIL).

## Discussion

Many miRNAs have been identified as biomarkers in various cancers. In our previous study, we confirmed that miR-375 played a role as a tumor suppressor in the pathogenesis of cervical cancer (21). We further validated that the relative expression levels of miR-375 and miR-424 in cervical exfoliated cells were significantly lower in high-grade CIN and invasive cervical cancer tissues than in low-grade CIN and normal cervical tissues, and miR-375 and miR-424 detection showed significantly higher sensitivity and NPV and comparable specificity and PPV compared with cytology in identifying high-grade CIN in the hospital population, suggesting the potential value of miR-375 and miR-424 detection positivity in triaging HPV-positive women for colposcopy.

In this study, we utilized residual cervical exfoliated cell samples from 10,951 women who had participated in a cervical cancer screening program to detect the expression of miR-375 and miR-424. In the 9,972 eligible women aged 25–65, the expression levels of miR-375 and miR-424 decreased along with the severity of cervical lesions. We further determined the cutoff values of two miRNAs in identifying high-grade CIN in the general population. According to the maximal Youden index, the cutoff value of miR-375 for the diagnosis of CIN2+ was 0.999 × 10^-3^. This cutoff value was close to 0.965 × 10^-3^, which was obtained from the hospital population in our previous study (17), and confirmed the stability of miR-375 detection, suggesting that miR-375 detection is also feasible for triaging HPV-positive women in the general population.

We then assessed the efficacy of miR-375 for identifying CIN3+ and CIN2+ in the HPV-positive population. When cytology (≥ASC-US) was used as a control, miR-375 detection showed similar sensitivity, specificity, PPV, and NPV for identifying high-grade CIN but had significantly higher sensitivity and NPV for identifying CIN2+. The CIN3+ immediate risk of miR-375 positivity among HPV-positive women was 19.8%, which reached the clinical action threshold for colposcopy recommended by the 2019 ASCCP guidelines (8), suggesting that the efficacy of miR-375 detection as a clinical action threshold for colposcopy is equivalent to that of cytology (≥ASC-US) in HPV-positive women. In the 2019 ASCCP guidelines, expedited treatment or colposcopy was recommended for HPV-positive/cytology-ASC-H women with an unknown previous screening history. Thus, we compared the efficacies between miR-375 and cytology (≥ASC-H) for expedited treatment or colposcopy and found that miR-375 positivity showed higher sensitivity and NPV but significantly lower specificity and PPV than cytology (≥ASC-H). The use of ASC-H as the clinical action threshold for expedited treatment or colposcopy in HPV-positive women is a new option in the 2019 ASCCP guidelines (8). The advantage of expedited treatment is the reduction in the number of colposcopy procedures in HPV-positive/ASC-H women, but most of them may be overtreated because the immediate CIN3+ risk of HPV-positive/ASC-H was only 26% in a study supporting the 2019 ASCCP guidelines (22). In addition, the much lower sensitivity of cytology (≥ASC-H) than that of ASC-US in our study suggests that some high-grade CINs are probably missed.

The 2015 ASCCP Interim Guidance recommended that the primary HPV test with HPV16/18 genotyping be used for cervical cancer screening, where HPV16/18-positive women are referred for colposcopy and HR12-positive women are triaged by cytology (6). For women who are HR12-positive, the 2019 ASCCP guideline raises the clinical action threshold for colposcopy from previous cytology (≥ASC-US) to current cytology (≥ASC-H). In this study, we found that the efficacy of miR-375 positivity for predicting CIN3+/CIN2+ was comparable to that of cytology (≥ASC-US) in HR12-positive women. However, when cytology (≥ASC-H) was used as a control, the sensitivity of miR-375 positivity was equivalent (predicting CIN3+) or increased (predicting CIN2+), while the specificity and PPV were significantly lower. Compared with cytology (≥ASC-US), cytology (≥ASC-H), as the threshold, can reduce the number of colposcopies but needs to be premised on the workability of the standard screening interval and good compliance of the examinee. However, in developing countries, cervical cancer screening with regular intervals is usually difficult to realize, so it is still very important to avoid missing as many high-grade lesions as possible in each round of screening. Adopting cytology (≥ASC-US) as the clinical action threshold for colposcopy in HPV-positive women may be more reasonable in developing countries. In HPV16-positive women, the sensitivity and NPV of miR-375 positivity for identifying CIN3+ and CIN2+ were higher, but the specificity and PPV were lower when cytology (≥ASC-H) or cytology (≥HSIL) was used as a control. However, the immediate CIN3+ risk of miR-375 positivity was 43.5%, which achieved the threshold for expedited treatment or colposcopy recommended by the 2019 ASCCP guideline (8), suggesting that miR-375 detection can also be used for women undergoing primary HPV16/18 genotyping screening.

## Conclusion

In summary, in HPV-positive or HR12-positive women, miR-375 detection had a similar efficacy as cytology (≥ASC-US) for identifying high-risk CIN and can be considered the clinical action threshold for colposcopy. In HPV16-positive women, miR-375 positivity had higher sensitivity and NPV than cytology (≥ASC-H) and cytology (≥HSIL), and the immediate CIN3+ risk achieved the risk threshold for expedited treatment or colposcopy recommended by the 2019 ASCCP guidelines. Since miRNA detection is a nonmorphological examination with objective results, miRNA detection, as a triage for primary HPV-positive women, avoids dependence on cytologists, and it is easier to train qualified laboratory technicians than qualified cytologists in developing countries, The detection of miR-375 in cervical exfoliated cells may be an optional method for triaging primary HPV-positive women in population-based cervical cancer screening in developing countries and regions where cytologists are insufficient.

## Data Availability Statement

The datasets presented in this study can be found in online repositories. The names of the repository/repositories and accession number(s) can be found below: https://github.com/chengxd1141/MiR-375-for-triaging-HPV-positive-women.

## Ethics Statement

The studies involving human participants were reviewed and approved by the Ethics Committee of Women’s Hospital, School of Medicine, Zhejiang University. The patients/participants provided their written informed consent to participate in this study.

## Author Contributions

XC and XW contributed to the conceptualization of this study. XX and QW contributed to the methodology. WL and LW performed the validation. QW and LW performed the formal analysis. XZ performed the investigation. XC contributed to the resources. QT and FW performed the data curation. QW contributed to writing—original draft preparation. XC and XW contributed to writing—review and editing. NS took charge of visualization. LQ supervised the study. XX participated in project administration. XC, XW, QW, and NS participated in funding acquisition. All authors contributed to the article and approved the submitted version.

## Funding

This work was supported by the Key Research and Development Project of Zhejiang Province (2020C03025), the Scientific Research Fund of Zhejiang Provincial Education Department (Y201840210), the National Natural Science Foundation of China (81974403), the Molecular Typing and Precision Control of Cervical Cancer Based on Proteomics Feature (2016YFC0902900), and Zhejiang Provincial Center for Diagnosis and Treatment of Uterine Malignant Tumor.

## Conflict of Interest

The authors declare that the research was conducted in the absence of any commercial or financial relationships that could be construed as a potential conflict of interest.

## Publisher’s Note

All claims expressed in this article are solely those of the authors and do not necessarily represent those of their affiliated organizations, or those of the publisher, the editors and the reviewers. Any product that may be evaluated in this article, or claim that may be made by its manufacturer, is not guaranteed or endorsed by the publisher.
