# Supplementary material for: The Value of MicroRNA-375 Detection for Triaging Primary Human Papillomavirus Positive Women: A Cross-Sectional Study in a General Population
Source: Front Oncol. 2021 Oct 28;11:771053. doi: 10.3389/fonc.2021.771053 (PMC8581639; doi:10.3389/fonc.2021.771053)
Supplement: Supplementary file 1 [file DataSheet_1.pdf]

Supplementary Table 1. Primers for reverse transcription (RT) and quantitative real-time polymerase chain reaction (qPCR)

Primers	Sequences
**Primers for RT**	
miR-375-RT	5'-GTCGTATCCAGTGCAGGGTCCGAGGTATTCGCACTGGATACGACTCACGC-3'
miR-424-RT	5'-GTCGTATCCAGTGCAGGGTCCGAGGTATTCGCACTGGATACGACTTCAAA-3'
U6-RT	5'-AACGCTTCACGAATTTGCGT-3'
**Reverse primers for qPCR**	
common reverse primer*	5'-GTGCAGGGTCCGAGGT-3'
reverse primer for U6	5'-AACGCTTCACGAATTTGCGT-3'
**Forward primers (FP) for qPCR**	
miR-375-FP	5'-AGCCGTTTGTTCGTTCGGCT-3'
miR-424-FP	5'-CGAAGCAGCAGCAATTCATG-3'
U6-FP	5'-CTCGCTTCGGCAGCACA-3'

* MiR-375 and miR-424 shared the same common reverse primer.
